# The use of both internal thoracic arteries for coronary revascularization increases the estimate of post-operative lower limb ischemia in patients with peripheral artery disease

**DOI:** 10.1186/s13019-020-01315-8

**Published:** 2020-09-25

**Authors:** Linda Renata Micali, Massimo Bonacchi, Daniel Weigel, Rosie Howe, Orlando Parise, Gianmarco Parise, Sandro Gelsomino

**Affiliations:** 1 Department of Cardiothoracic Surgery, Cardiovascular Research Institute Maastricht - CARIM, Maastricht University Medical Center, 6229 ER Maastricht, The Netherlands; 2 Careggi Hospital, Florence, Italy

**Keywords:** Coronary disease, Peripheral artery disease, Limb ischemia

## Abstract

**Background:**

Patients with a history of peripheral arterial disease (PAD) undergoing coronary artery bypass grafting (CABG) exhibit higher rates of complications. There are conflicting data on the survival benefits for bilateral thoracic artery (BITA) grafting compared with left internal thoracic artery (LITA) CABG in patients with PAD. The aim of the study was to explore the influence of the use of BITA grafts vs. LITA for CABG on post-operative acute lower limb ischemia (ALLI) and main post-operative complications in patients with concomitant PAD.

**Methods:**

We used a propensity-score (PS) based analysis to compare outcomes between the two surgical procedures, BITA and LITA. The inverse probability of treatment weighting PS technique was applied to adjust for pre- and intra-operative confounders, and to get optimal balancing of the pre-operative data. The primary outcome was the estimate of postoperative ALLI. Secondary outcomes included overall death and death of cardiac causes within 30 days of surgery, stroke and acute kidney disease (AKD).

**Results:**

The study population consisted of 1961 patients. The LITA procedure was performed in 1768 patients whereas 193 patients underwent a BITA technique. The estimate of ALLI was 14% higher in the BITA compared to the LITA (*p* < 0.001) group. Thirty-day mortality, cardiac death, occurrence of stroke and AKI did not differ significantly between the groups.

**Conclusions:**

The use of both ITAs led to a significant increase in ALLI. This result was most likely caused by the complete disruption of the ITA collateral providing additional blood supply to the lower extremities. Based on our data, BITA should be used with extreme caution in PAD patients. Further research on this topic is necessary to confirm our findings.

## Introduction

The prevalence of coronary artery disease (CAD) among patients with peripheral arterial disease (PAD) is as high as 60% [1, 2], and 10–30% of patients who are treated for CAD by surgical interventions suffer from PAD [3, 4].

Patients with a history of PAD who undergo coronary artery bypass grafting (CABG) exhibit higher rates of complications [5]. Therefore, the selection of an appropriate surgical technique is of the outmost importance [6]. In particular, the choice of revascularization strategy and the graft selection are key factors for the success of procedures in these patients.

While large studies have reported a survival benefit for bilateral thoracic artery (BITA) grafting compared with left internal thoracic artery (LITA) CABG [7, 8] there is conflicting data published from other studies [9–14]. There is even more uncertainty into the choice of coronary revascularization strategy in the case of PAD patients. As a result, no precise guidelines on graft selection exist for this important subpopulation of patients, and the choice of using one or two thoracic arteries is left to the surgeon’s judgement.

We explore the influence of the use of double thoracic artery grafts on post-operative limb ischemia (ALLI) and on the main post-operative complications in patients with PAD.

## Methods

Ethical Committee approval was waived due to the retrospective analysis nature of the study according to National Laws regulating observational retrospective studies (Italian law nr.11960, released on 13/07/2004).

All consecutive patients with PAD undergoing isolated CABG between 1997 and 2019 were included in the analysis. Subjects with previous or concomitant vascular surgery were excluded from the study. A total of 1961 subjects represents the final study population.

The patients were divided into two groups based on the use of the LITA (*n* = 1768) or BITA (*n* = 193) technique.

Clinical PAD was defined as patients who, at the time of CABG including one or more of the following criteria: claudication with exertion or rest; documented aortoiliac occlusive disease, documented abdominal aortic aneurysm or documented noninvasive testing, including ankle-arm index (AAI).

To ascertain high-quality reporting, we followed the guidelines in the article Strengthening the Reporting of Observational Studies in Epidemiology (STROBE), specifically modified for propensity-score research. The list of items addressed are shown in the Supplementary Material.

The main endpoint was ALLI defined as any ischemic event of the arterial system of the lower limbs following the Inter-Society Consensus for the Management of Peripheral Arterial Disease (TASC II) [15]. The secondary endpoints were: (a) death, being defined as death of any cause occurring in or outside the hospital setting within 30 days after the intervention or > 30 days if within the same hospitalization period, (b) cardiac death being defined as death attributed to cardiac events; (c) stroke characterized as a neurological deficit lasting > 24 h, being CT or MRI confirmed and assessed by a neurologist; and (d) acute kidney disease defined as a post-operative creatinine level of > 200 mmol/L or the demand for dialysis.

### Surgical details

The LAD and its branches were the target for the skeletonized in situ LITA. Whereas, the target for the skeletonized in situ RITA of the remaining components of left coronary artery were selected according to the following criteria: in patients with two branches allowing for grafting, the primary target was the branch with a bigger perfusion area; if both branches did not differ in terms of dimension of perfusion area, the target for the RITA grafting was the distal branch and the other vessel was revascularized with composite, sequential ITA grafts or alternatively other conduits. The surgeons performing the procedure kept the pleurae closed in order to preserve the communicating bifurcation of the ITAs to the chest wall and to protect the branches of the pericardiophrenic artery. During the procedure, the free flow of the ITAs was assessed. As it was > 50 mL/min in all cases, all ITAs were utilized.

### Statistical analysis

Propensity score (PS) inverse-probability of treatment weighting estimation was used [16–19]. Balance was tested either graphically or by means of balance tables. In the model, absolute standardized mean differences (ASMD) were calculated using a cutoff of less than 0.20 for bias statistics. The average treatment effect among the treated (ATT) was chosen as the causal effect estimand of stroke. R software v. 3.6.1 packages were used for analysis. More details can be found in the Supplemental Material (Statistical notes).

## Results

### Assessment of balance by boxplots

**Fig. 1:**
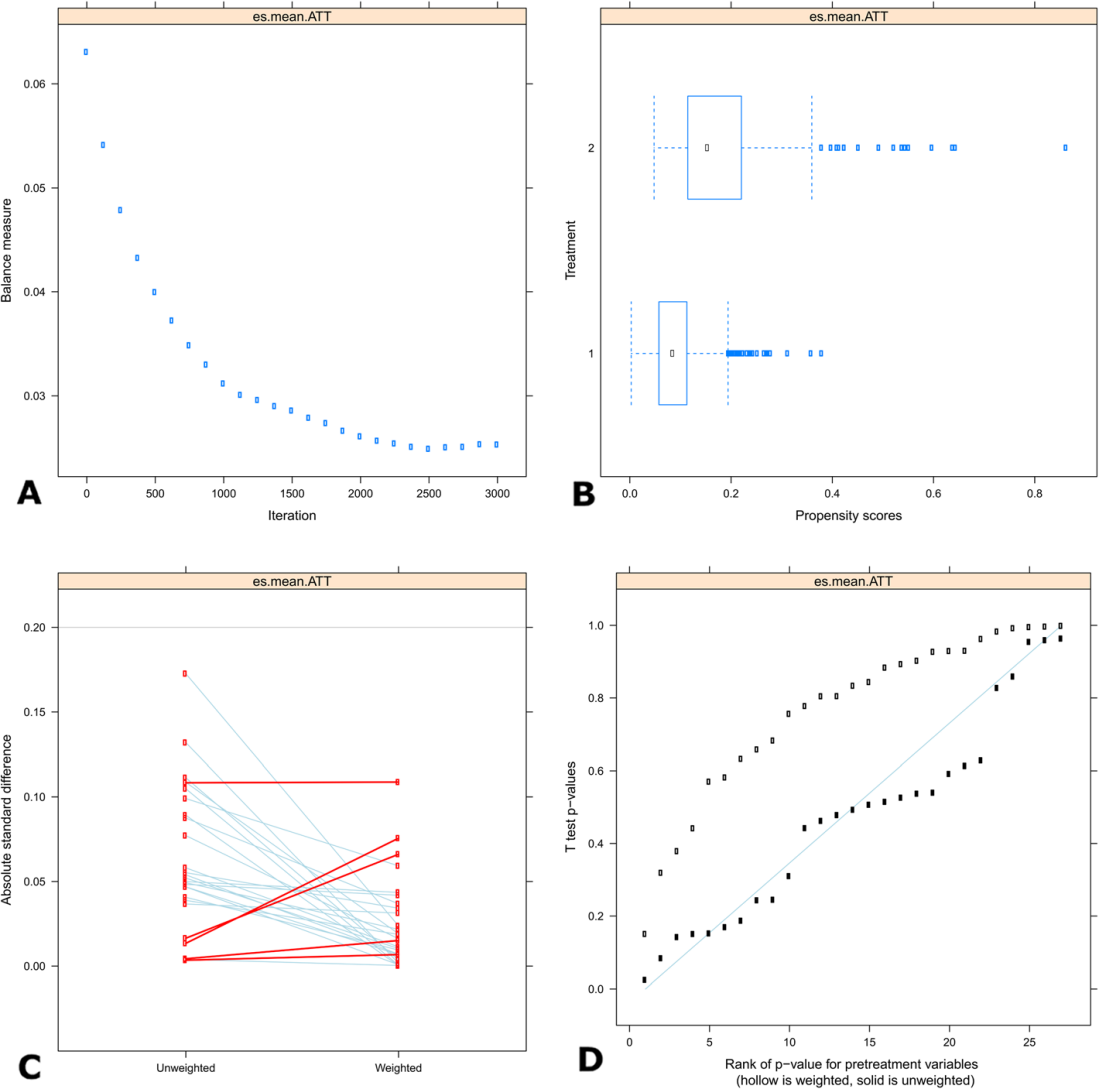
**a**. Optimize plot. The plot is a graphical display of the balance criteria as a function of the GBM iteration. The graph demonstrates that a favorable balance was achieved with 1000 iterations and that such a balance was worse with a higher number of iterations that, thus were unnecessary. **b**. Overlap Assessment. The figure presents two sets of box plots of PS distributions. An overlap between the groups is desirable meaning that there are no values of the pretreatment variables that occur only in one of the treatment conditions. However, there are no specific rules for what constitutes sufficient overlap so, in doubtful cases the combination of the overlap plot and the balance table are used. In our model there is a good overlap between the two boxplots. **c**. Standardized Effect Size plot. It assesses the balance of pretreatment variables before and after weighting. It shows the maximum pairwise ASMDs. The ASMDs cutoff for defining unbalanced variables was 0.20. The light blue line represents pretreatment covariates for which the maximum pairwise ASMD reduced after weighting. The red lines mean the pretreatment covariates for which the maximum pairwise ASMD increased after weighting. A good balance is obtained when, after weighting in the majority of variables the ASMDs are < 0.20 and there is a prevalence of light blue lines. In our model, after weighting the pretreatment variables, the balance showed excellent results with all ASMD values being well below 0.2. **d**. Quantile-quantile (Q-Q) plot. This plot also assesses the balance of pretreatment variables. In the plot, the Kolmogorov-Smirnov p-value is plotted against the rank of *p*-value for pretreatment variables. Along a 45-degree fitting line, open symbols represent weighted covariates and solid symbols represent unweighted covariates. A good balance is obtained when open symbols lie close, below or above, the 45-degree line. After weighting, in our model, all covariates were well above the reference line

We found that a good balance was achieved with 1000 iterations **(**Fig. 1a**)**. The PS distribution **(**Fig. 1b**)** for the LITA group ranged from 0.0 to 0.2, whereas for the BITA group, the distribution ranged from 0.05 to 0.36, showing a substantial overlap among the two groups. The absolute standardized difference (ASMD) **(**Fig. 1c**)** without weighting was below 0.2 in all cases with the highest equaling − 0.17. After the weighting procedure, the highest value was 0.11; therefore, the ASMD was much lower than 0.2 for all cases. The Q–Q plot **(**Fig. 1d**)** before weighting already showed a good balance with all plots being close to the diagonal. After weighting, nearly all of the *p*-values were located well above the diagonal, thereby illustrating a good balancing of the characteristics among the two groups.

### Assessment of the balance table

The balance table (Table 1) showed that the highest standard effect size (std.eff.sz) differences before weighting were found for two vessel disease (− 0.17), three vessel disease (0.13), previous percutaneous transluminal coronary angioplasty (PTCA) (0.11), dialysis (0.11), stroke history (− 0.11), carotid stenosis > 50% (0.1), and left ventricular ejection fraction (LVEF) (0.1). After weighting, the highest std.eff.sz was 0.11 (dialysis).

**Table 1:** Balance of patient characteristics (*n* = 1961)

	Unweighted			ATT Weighted		
	LITA ***n*** = 1768	BITA ***n*** = 193	**std.eff.sz**	LITA ***n*** = 1768	BITA ***n*** = 193	**std.eff.sz**
Age	70.44	70.48	0	70.6	70.48	−0.02
Gender (Male)	0.81	0.77	−0.09	0.79	0.77	−0.04
Hypercholesterolemia	0.65	0.68	0.05	0.68	0.68	−0.01
Hypertension	0.79	0.81	0.05	0.81	0.81	0.01
COPD	0.1	0.12	0.05	0.11	0.12	0.04
Diabetes	0.4	0.42	0.05	0.41	0.42	0.02
Diabetes with Insulin	0.18	0.18	0	0.18	0.18	−0.01
Dialysis	0.02	0.04	0.11	0.02	0.04	0.11
AKD	0.1	0.09	−0.05	0.08	0.09	0.04
Active Smoke	0.55	0.57	0.04	0.57	0.57	0.01
Stroke History	0.06	0.04	−0.11	0.04	0.04	0
TIA History	0.07	0.09	0.06	0.08	0.09	0.03
Carotid Stenosis > 50%	0.14	0.18	0.1	0.16	0.18	0.06
Antiplatelet drugs	0.3	0.27	−0.08	0.28	0.27	−0.02
LVEF	50.16	51.28	0.1	51.27	51.28	0
NYHA ≥3	0.26	0.26	0	0.26	0.26	0
IABP	0.02	0.02	−0.04	0.01	0.02	0.02
Previous PTCA	0.37	0.42	0.11	0.42	0.42	0.02
LAD Stenosis	0.93	0.94	0.05	0.94	0.94	0.01
MCA Stenosis	0.38	0.4	0.06	0.4	0.4	0.01
Three vessels disease	0.71	0.76	0.13	0.76	0.76	0
Two vessels disease	0.29	0.22	−0.17	0.23	0.22	−0.02
Pulmonary Hypertension	0.08	0.08	0.01	0.06	0.08	0.08
REDO	0.15	0.12	−0.09	0.12	0.12	0
Surgery						
Elective	0.63	0.65	0.04	0.66	0.65	−0.03
Urgency	0.05	0.06	0.02	0.04	0.06	0.07
Emergency	0.32	0.3	−0.05	0.3	0.3	0

Abbreviations: *LITA* Left internal thoracic artery; *BITA* Bilateral internal thoracic artery; *COPD* Chronic obstructive pulmonary disease; *AKD* Acute kidney disease; *TIA* Transient ischemic attack; *LVEF* Left ventricular ejection fraction; *NYHA* New York heart association (functional class); *IABP* Intra-aortic balloon pump; *PTCA* Percutaneous transluminal coronary angioplasty; *LAD* Left anterior descending; *MCA* Main coronary artery. *REDO* Re-operative surgery

### Unweighted early outcomes

The early crude outcomes by the procedure performed are shown in Table 2. In the unweighted population, there was a higher estimate of ALLI in the BITA group compared to the LITA group (34.7% vs. 19.3%, *p* < 0.001). No other differences were detected between the two groups. There were 69 sternal wound infection, 33 (17%) in the BITA and 36 (2.0%) in the LITA Groups, respectively (*p* < 0.001).

**Table 2:** Post-operative data

	Overall	LITA	BITA	***p***
	***n*** **= 1961**	***n*** **= 1768**	***n*** **= 193**	
ALLI	408 (20.8)	341 (19.3)	67 (34.7)	< 0.001
ALLI treatment				
Thrombectomy	89 (21.8)	74 (21.8)	15 (22.4)	
Fasciotomy	67 (16.4)	56 (16.4)	11 (16.4)	
Endarterectomy	10 (2.5)	5 (1.5)	5 (7.5)	
Percutaneous intervention	126 (30.9)	108 (31.7)	18 (26.8)	
Surgery	83 (20.3)	68 (19.8)	15(22.4)	
Others/Unknown	33 (8.1)	30 (8.8)	3 (4.5)	
Death	67 (3.4)	59 (3.3)	8 (4.1)	0.71
Cardiac Death	31 (16)	26 (1.5)	5 (2.6)	0.38
Stroke	121 (6.2)	111 (6.3)	10 (5.2)	0.56
AKD	276 (14.1)	252 (14.3)	24 (12.4)	0.561

Values are expressed as n (%) or median [interquartile range]. Abbreviations: *LITA* Left internal thoracic artery; *BITA* Bilateral internal thoracic artery; *ALLI* Post-operative limb ischemia; *AKD* Acute kidney disease

### Main endpoint and secondary endpoints in the weighted population

**Fig. 2:**
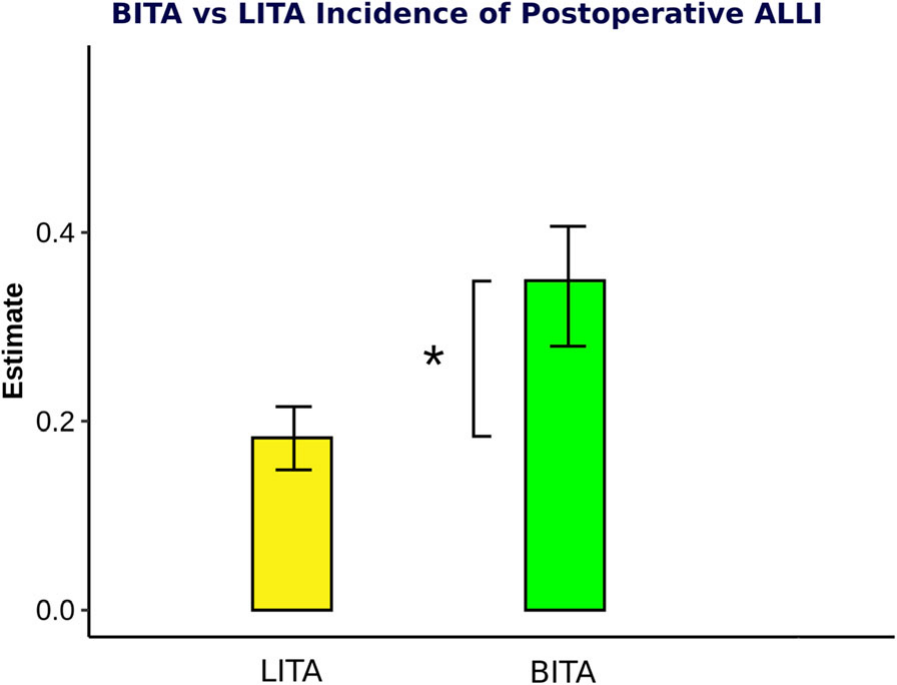
The estimate and 95% CI of post-operative ALLI in employing LITA or BITA. *p < 0.001(df.0.14 [0.07–0.22]

After weighting, the estimate of ALLI was higher at 14% in the BITA compared to the LITA group. (+ 14% compared to LITA (95% Confidence Interval [CI] 7.0–22.0%) *p*-value < 0.001) **(**Fig. 2**)**. The weighted secondary endpoints are shown in Table 3**.** Neither the occurrence of death (+ 1.3% vs. LITA (95% CI − 1.6 to 4.2%) *p*-value = 0.37) nor cardiac death (+ 1.4% vs. LITA (95% CI − 0.9 to 3.7%) *p*-value = 0.23) significantly differed between the LITA and BITA groups. The comparison of the estimate of stroke (− 0.3% vs. LITA (95% CI − 3.7 to 2.9%) *p*-value = 0.81) and the appearance of post-operative acute kidney disease (AKD) (+ 1.4% vs. LITA (95% CI − 3.0 to 6.0%) *p*-value = 0.57) were not significantly different between the two groups.

**Table 3:** ATTs Treatment Group Means

	Estimate	95% CI	***p***-value
**Death**			
ATT Weighted			
(Intercept)	0.028	0.019, 0.036	< 0.001
BITA	0.013	−0.016, 0.042	0.37
**Cardiac Death**			
ATT Weighted			
(Intercept)	0.011	0.006, 0.017	< 0.001
BITA	0.014	−0.009, 0.037	0.23
**Stroke**			
ATT Weighted			
(Intercept)	0.055	0.044, 0.067	< 0.001
BITA	−0.003	−0.037, 0.029	0.81
**AKD**			
ATT Weighted			
(Intercept)	0.11	0.09, 0.12	< 0.001
BITA	0.014	−0.03, 0.06	0.58

Note: The intercept represents the estimate of the respective endpoint in the LITA group. The BITA group estimate illustrates the change in estimate, compared to the intercept, expressed in percent increase or decrease

Abbreviations: *CI* Confidence interval; *LITA* Left internal thoracic artery; *BITA* Bilateral internal thoracic artery; *AKD* Acute kidney disease

## Discussion

The main aim of this study was to analyze the influence of two different CABG techniques involving the use of single or double thoracic arteries on the occurrence of post-operative ALLI in patients with PAD. The importance of PAD as a predictor of reduced long-term survival and increased risk for complications following CABG has been sufficiently documented [4, 20–23]. Any final conclusion is far from being drawn as to whether a specific technique may help in improving the outcomes and, more specifically, whether the use of the double mammary artery technique is the right choice for these patients when considering its established advantages [9–13].

The main finding of our paper was the significantly higher ALLI estimate in patients receiving a BITA graft (+ 14% (7–22%)). To the best of our knowledge, our report included the largest collection of patients with PAD undergoing CABG surgery, focused on the occurrence of post-operative ischemia related to bilateral ITA harvesting.

To date, few case reports have highlighted this potential complication after CABG [24–30]. Hirose et al. [31] presented 13 patients with concomitant aortoiliac occlusive disease with collateral circulation via unilateral or bilateral ITA(s). Furthermore, Ben-Dor et al. [29] observed a collateral flow from one or both internal mammary arteries to an occluded or stenotic iliac artery in 15 patients undergoing diagnostic coronary angiography.

Nakatsu et al. [23] found, in 136 patients, a significant increase in post-operative percutaneous transluminal angioplasty but not in surgical revascularization procedures after BITA-CABG procedures. The development of postoperative ALLI is highly predictive of adverse events and mortality [32] but, despite this, there is no robust, controlled analysis on expected outcomes in patients with ALLI after CABG surgery related to the technique employed.

Our data provided clear evidence for the first-time for the increased estimate of ALLI employing BITA in a large patient population.

Chronic aortoiliac occlusive disease induced the development of several collateral arterial pathways that derive from a common embryologic origin [33]. These pathways can be systemic–systemic or visceral–systemic. The Winslow pathway [34, 35] is a systemic–systemic pathway that links the internal thoracic artery, superior epigastric artery, inferior epigastric artery, and external iliac artery and is prominent in aortoiliac obstructive disease [36]. This pathway provides more than 40% of the arterial flow needed to perfuse the lower extremities [37]. ITAs are also involved into the systemic–systemic intercostal artery collateral pathway being connected, through the superior and inferior abdominal epigastric arteries, to the external iliac artery through a network of lumbar and iliolumbar arteries [35].

Parashara et al. reported [38] a patient where the left ITA supplied the left external iliac artery. The main visceral–systemic collateral network includes the mesenteric arteries, the artery of Moskowitz, and the marginal artery of Drummond connected with the internal iliac artery through the superior rectal artery branches and the middle rectal artery branch, with both originating from the inferior mesenteric arteries. These vessels can also communicate with the systemic circulation through branches of the femoral and external iliac arteries. Other visceral–systemic pathways involve the gonadal arteries, arising from the renal arteries or from the aorta [35].

In the case of PAD, the relative contribution of each of these collateral pathways in preserving limb perfusion is unknown [24] and depends on the location and severity of the aortic occlusion. The Winslow pathway is the most important flow supply to the lower extremities, and cases of lower limb ischemia have been reported following ITA harvesting in patients with aortoiliac occlusive disease [39–41]. Therefore, the pre-operative identification of arterial collateral pathways is recommendable in patients with PAD.

Conventional angiography may fail to reveal this collateral pathway, and lower extremity run-off is better visualized during subclavian and/or internal thoracic arteriography than during aortography [42]. Routine visualization of the ITAs before bypass surgery is usually considered unnecessary. Selective angiography of the ITA requires a relatively long period of exposure time, and may result in infrequent complications, such as dissection or embolization with subsequent cerebrovascular ischemia [31]. However, selective ITA angiography can reveal significant proximal subclavian artery stenosis and, when it is performed, allows the visualization of intra-abdominal anastomoses in the presence of highly developed superior and inferior epigastric arteries and of large ITAs [43], which has been directly related to the severity of aortoiliac disease [37, 44].

However, as complication rates are low, and significant findings are often found [45, 46], selective ITA injection is advisable in potential candidates for CABG, in subjects with aortoiliac occlusive disease, subjects with a history suggestive of PAD, subjects where clues of such a condition were available from the patient’s medical history, subjects with an absent or weak femoral pulse, and in subjects with an abnormal AAI or absolute ankle perfusion pressures. CT angiography methods can also show the opacification of these arteries with a contrast agent, yielding a reliable visualization of the Winslow pathway [30].

Duplex scanning and directional Doppler are another option to reveal this collateral pathway, demonstrating the reversal of blood flow in the inferior epigastric artery [36].

Our findings seem to discourage the use of bilateral ITA in PAD patients. The higher estimate of ALLI in our BITA patients indirectly confirms that the disruption of the left and right collateral Winslow routes would significantly increases the risk of severe post-operative limb ischemia. This acute interruption might not allow the activation of other secondary pathways as it occurs in chronic occlusions. In addition, with a large caliber of the ITAs during the intra-operative harvesting, the diameter of the ITA may hint towards collateralization via the ITAs [28], and, in this case, the preservation of at least one ITA and the use of alternative conduits is advisable. The cardiac surgeon should also investigate whether the patient had previous abdominal surgery as this collateral pathway could be accidentally ligated during renal and scrotal interventions. Post-operatively, we recommend a close follow-up with screening for the clinical signs of reduced limb perfusion in combination with ABI-measurements [47]. In the case of patients with a stable angina, aorto-iliac revascularization before CABG is an option to be evaluated [31]. The advantages are that, after revascularization of the leg, the ITAs can be used, and the presence of large ITAs becomes a benefit for the patient.

The secondary outcome analysis demonstrated no difference in either short-term overall or cardiac-related mortality. Our data is in accordance with a small study among patients with PAD undergoing either LITA or BITA procedures that did not find any significant difference in in-hospital mortality [23]. Another single center study, analyzing the effect of BITA against LITA augmented with radial artery grafts, in a cohort of PAD patients, demonstrated no significant difference with regard to the 30-day survival [48]. A meta-analysis on the topic suggested an increased short-term mortality, which was attributed to a higher risk for post-operative complications and, in particular, deep sternal wound infections [12]. Our findings were not in accordance with this data.

Although not specifically evaluating the PAD subpopulation, a recent, large RCT comparing LITA vs. BITA found no difference in the long-term mortality [10]. Those results contradicted the many observational studies that suggested a BITA superiority [12, 13, 49]. As Gaudino et al. [50] described, results speaking in favor of the BITA approach may be attributed to non-measured confounders rather than to a true superiority of the procedure. The evidence is high that the short-term survival is not significantly different among the two procedures and is generally rather low (a 30-day mortality of 1.2%) [51–53]. Whether the mortality rates begin diverging after 5–10 years, as stated by Ryan et al. [54], remains a matter of debate. Warranting further research is the finding by Efird et al. [55], who noticed that black patients with PAD undergoing CABG had higher mortality rates compared to a matched white population.

The occurrence of stroke did not significantly differ between the subgroups during the first 30 days post-operatively. The estimate of stroke in our study population was approximately 5%; which is much higher than the 2% estimate for stroke reported in the regular CABG population [56]. This could be attributed to PAD being an independent risk factor for stroke development following CABG, thereby posing a generally higher risk for cerebrovascular accidents in our patient population [21, 57]. Nonetheless, we could not establish a link between the applied grafting method and stroke. Bearing in mind the therapy recommendations for patients at risk for periprocedural stroke, for instance antiplatelet therapy and atrial fibrillation screening [58], from our findings there seems not to be a contraindication for the use of both ITAs with respect to the causation of stroke. Finally, this study did not show a significant discrepancy in the development of post-operative acute kidney disease (AKD) among the two subgroups. This is an important finding, as even minor post-operative changes in kidney functioning are associated with a highly increased risk for development of end-stage renal disease as well as mortality [59–61]. Generally, PAD patients are at risk to develop AKD not only because this patient group has a high prevalence of diabetes but also because generalized atherosclerosis is an independent risk factor for AKD development [62]. The high prevalence of renal artery stenosis of 23–42% in PAD patients further substantiates the relationship between PAD and renal disease [1]. This indicates that PAD patients are at risk as it was demonstrated that pre-intervention kidney disease is an independent predictor of worse outcomes and increased mortality following CABG [20]. In line with our findings Nakatsu et al. [63] demonstrated no statistically important effect from LITA vs. BITA procedures on the long-term survival in patients with pre-operative end-stage renal disease. Consequently, with respect to the hazard inherent to cardiac surgeries, BITA would appear equally as safe as other CABG methods.

### Limitations

This study has some limitations that need to be highlighted. First, the paper carries the inherent limitations of retrospective observational studies. Nonetheless, the PS multifactor method allowed us to have a population with the absence of baseline unbalances and, at the same time to retain all the patients, leading to a large population study. Second, no stratification was performed between the different types of PAD. In consequence, it cannot be excluded that the BITA group population had a higher estimate of aortoiliac stenosis, thereby biasing the measured outcome. Also, it was not possible to test whether the contribution of collateral pathways in preserving limb perfusion depends on location and severity of occlusions and whether there was any difference between aortic and iliac artery occlusions.

Moreover, the ITA diameters, being an important indicator for collateralization, were not documented as the patients did not undergo routine direct ITA visualization. Pre- and post-operative images would be a valuable source for further evaluation of the effect of BITA on ALLI.

Third, not all patients Underwent ankle-arm index evaluation. Fourth whereas we identified cases on the basis of the definition of ALLI, limited data are available beyond this definition regarding details, severity, or timing of each patient’s ALLI.

Fifth, cardiac embolization, as a possible reason for ALLI, was not specifically evaluated. Sixty, our study did not account for the adherence to therapy and the evaluation of the risk factor profile in the respective study populations. This may have added a bias to our results as the importance of risk factors and pharmacological treatment should not be underrated. Seventh, no comparison was carried out with patients undergoing limb revascularization before CABG. However, this was beyond the aim of this study and will be the object of further research. Eighth, we did not perform an external validation, and this might affect the generalizability of our findings.

Finally, it is possible that patients with more severe PAD were selected for BITA to avoid.

wound healing issues following saphenous vein harvesting. This might have introduced a bias and, since we do not have information regarding the severity of PAD, it is was impossible to address this problem of residual confounding in the statistical analysis.

## Conclusions

The usage of both ITAs increased the development of ALLI in patients with PAD. Additional prospective trials are necessary in order to confirm our findings.

## Supplementary information

**Additional file 1.**

## Data Availability

The datasets used and/or analyzed during the current study are available from the corresponding author on reasonable request.

## Abbreviations

AAI: Ankle-arm index
AKD: Acute kidney disease
ALLI: Acute lower limb ischemia
ASMD: Absolute standardized mean difference
BITA: Bilateral internal thoracic artery
CABG: Coronary artery bypass grafting
CAD: Coronary artery disease
ITA: Internal thoracic artery
LITA: Left internal thoracic artery
PAD: Peripheral arterial disease
PS: Propensity score
PTCA: Percutaneous transluminal coronary angioplasty
