# Supplementary material for: The use of both internal thoracic arteries for coronary revascularization increases the estimate of post-operative lower limb ischemia in patients with peripheral artery disease
Source: J Cardiothorac Surg. 2020 Sep 25;15:266. doi: 10.1186/s13019-020-01315-8 (PMC7519572; doi:10.1186/s13019-020-01315-8)
Supplement: Supplementary file 1 — Additional file 1. [file 13019_2020_1315_MOESM1_ESM.docx]

**Supplementary Material**

| **Supplementary Table S1.** Full guidelines for reporting propensity score analysis, modified From the STROBE (STrengthening the Reporting of Observational studies in Epidemiology) Statement* | | | |
| --- | --- | --- | --- |
| **Section/topic** | **Item No** | | **Recommendation** |
| **Title and abstract** | **x** | 1 | Indicate the use of propensity analysis with a commonly used term in the title or the abstract |
|  | **x** | 2 | Provide in the abstract an informative and balanced summary of what was done and what was found |
| **Introduction** |  |  |  |
| Background/rationale | **x** | 3 | Explain the scientific background and rationale for the investigation being reported |
| Objectives | **x** | 4 | State specific objectives, including any prespecified hypotheses |
| **Methods** |  |  |  |
| Setting | **x** | 5 | Describe the setting, locations, and relevant dates, including periods of recruitment, treatment, follow-up, and data collection |
| Patient selection | **x** | 6 | Give the eligibility criteria, and the sources and methods of subject ascertainment and selection |
| Variables | **x** | 7 | Clearly define all outcomes, treatments, predictors. Give diagnostic criteria, if applicable |
| Data sources/ measurement | **x** | 8 | For each variable of interest, give sources of data and details of methods of assessment (measurement) |
| Bias | **x** | 9 | Describe how propensity score analysis was used to address bias |
|  | **x** | 10 | Describe any other methods to address potential sources of bias, e.g. sensitivity analysis |
| Sample size | **x** | 11 | Explain how the study size was arrived at |
| Statistical analyses | **x** | 12 | Describe all the analytic methods, including the propensity score methods, e.g. matching, weighting, stratification, or covariate adjustment using propensity score |
|  | **x** | 13 | Indicate the model used to estimate propensity score, e.g. logistic model, boosting (meta-classifiers), decision trees |
|  | **x** | 14 | State the variables included in the propensity score model |
|  | **x** | 15 | Explain the variable selection procedure for propensity score model |
|  |  | 16 | For propensity score matching: |
|  | **NA** | 16.1 | Explicitly state the matching algorithm and distance metric |
|  | **NA** | 16.2 | Indicate matching ratio (1:m matching) |
|  | **NA** | 16.3 | Indicate whether sampling with or without replacement was used |
|  | **NA** | 16.4 | Describe the statistical methods for the analysis of matched data |
|  | **NA** | 16.5 | Describe methods for assessing the comparability of baseline characteristics in the matched groups |
|  | **x** | 17 | For propensity score weighting, describe methods for assessing the comparability of baseline characteristics in the weighted groups |
|  |  | 18 | For propensity score stratification: |
|  | **NA** | 18.1 | Give the number of strata |
|  | **NA** | 18.2 | Describe methods for assessing the comparability of baseline characteristics in each stratum |
|  | **x** | 19 | Explain how assumption of propensity score analysis was examined |
|  | **x** | 20 | Explain how missing data were addressed, including missing data in propensity score estimation |
|  | **x** | 21 | If applicable, describe any methods used to examine subgroups and interactions |
|  | **x** | 22 | Describe any sensitivity analyses |
|  | **x** | 23 | Indicate the software used for analysis |
|  | **x** | 24 | If applicable, report the package used to create matched sample, e.g. GMATCH macro in SAS, MatchIt package®, Optmatch package ® |
| **Results** |  |  |  |
| Participants |  | 25 | Report numbers of participants at each stage of study: |
|  | **x** | 25.1 | sample size of patients potentially eligible |
|  | **x** | 25.2 | sample size of patients confirmed eligible and included |
|  | **x** | 25.3 | sample size of patients analyzed |
|  | **x** | 25.4 | for propensity score matching, sample size for each treatment group before and after matching |
|  | **x** | 26 | Explain reasons for exclusion at each stage |
|  | **NA** | 27 | Consider use of a flow diagram |
| Patient characteristics | **x** | 28 | Describe the distribution of baseline characteristics for each group before propensity score analysis |
|  |  | 29 | For propensity score matching, weighting, or stratification: |
|  | **NA** | 29.1 | Desc Describe the distribution of baseline characteristics in the matched/weighted groups or in each stratum |
|  | **NA** | 29.2 | Describe the results of the comparability of baseline characteristics, whether there are still systematic differences between treatment groups |
|  | **NA** | 30 | Indicate number of patients with missing data for each variable of interest, especially the variables used  in propensity score model |
| Outcome data | **x** | 31 | Report outcomes of each treatment group |
| Main results | **x** | 32 | Give propensity score analysis estimates and their precision, e.g. 95% confidence interval |
|  | **x** | 33 | If applicable, give unadjusted estimates and/or adjusted estimates and their precision, e.g. 95% confidence interval. Make clear which additional factors were adjusted for |
| Other analyses | **NA** | 34 | Report other analyses done, e.g. analyses of subgroups and interactions, and sensitivity analyses |
| **Discussion** |  |  |  |
| Key results | **x** | 35 | Summarize key results with reference to study objectives |
| Limitations | **x** | 36 | Discuss limitations of the study, taking into account sources of potential bias or imprecision |
|  | **x** | 37 | Discuss both direction and magnitude of any potential bias |
| Interpretation | **NA** | 38 | Discuss whether imbalance of baseline characteristics still exists, and give a cautious interpretation |
|  | **x** | 39 | Give a cautious overall interpretation of results considering objectives, limitations, multiplicity of analyses, results from similar studies, and other relevant evidence |
| Generalizability | **NA** | 40 | For propensity score matching, discuss the possibility and potential influence of incomplete matching, especially the studies in which the matched sample size is less than 50% |
| **Other information** |  |  |  |
| Funding | **x** | 41 | Give the source of funding and the role of the funders for the present study and, if applicable, for the original study on which the present article is based |

* von Elm E, Altman DG, Egger M, et al. The Strengthening the Reporting of Observational Studies in Epidemiology (STROBE) statement: guidelines for reporting observational studies. J Clin Epidemiol 2008;61(4):344-9.

Yao, X. I. *et al.* Reporting and Guidelines in Propensity Score Analysis: A Systematic Review of Cancer and Cancer Surgical Studies. *J Natl Cancer Inst* **109**, djw323, doi:10.1093/jnci/djw323 (2017)

<https://sites.duke.edu/xiaofeiwang/files/2016/12/Supplementary-Table-6.pdf>

Abbreviations: ASMD: Absolute Standardized Mean Difference; COPD: Chronic Obstructive Pulmonary Disease; AKD: Acute Kidney Disease; TIA: Transient Ischemic Attack; LVEF: Left Ventricular Ejection Fraction; NYHA: New York Heart Association (functional class); IABP: Intra-aortic Balloon Pump; PTCA: Percutaneous transluminal coronary angioplasty; LAD: Left Anterior descending; MCA: Main Coronary Artery.

**Statistical Notes (1-12)**

A generalized boosted model was employed for the propensity score estimation. We chose the average treatment effect among the treated (ATT) as the causal effect estimand. The ATT is the comparison of the mean patient outcomes, for instance, with the use of LITA compared with the mean outcome they would have had if they had instead been treated with BITA. The inverse probability of treatment weighting (IPTW) was employed to reduce the confounding due to the observed variables. The positivity assumption was assessed by testing the overlap in the true distribution of the observed pretreatment characteristics. The potential unmeasured confounders were tested by their relationship to the possible confounders within the treatment groups. We used generalized boosted models (GBM) to estimate the propensity score weights to fit separate GBMs, one for each group.

The Kolmogorov–Smirnov (KS) statistic was employed as a stopping rule to select the optimal iteration of GBM for use in estimating the PS weights. The balance of each group toward the others was checked either by the diagnostic plots or using a table. An optimize plot was employed as a graphical display of the balance criteria as a function of the GBM iteration. We tested the overlap using boxplots of the PS scores for the treatment aiming at a substantial overlap of the box plots with a great separation of the plots typically considered insufficient. We plotted the absolute standardized mean differences (ASMD) between the treatment groups on the pretreatment covariates, before and after weighting. Finally, we used the quantile–quantile plot to further check the balance. In this plot, the quantiles of the observed p-values to the quantiles of the uniform distribution (45-degree line) were displayed.

Ideally, in the independent tests in which the null hypothesis is true, the p-values will have a uniform distribution. In contrast, a severe deviation of the p-values below the diagonal suggests a lack of balance, and p-values running at or above the diagonal suggests that balance might have been achieved. For the bias statistics, ASMD values <0.20 were considered small.

The estimation of ATTs was implemented by fitting weighted regression models.

The weighted models yielded the estimates of ATTs along with 95% confidence intervals (and corresponding p-values) for assessing statistical significance.

Both propensity score adjustment and additional covariate adjustment was utilized referring to the year of surgery to obtain a robust estimates of the treatment effect.

R software v. 3.5.2 (R Foundation for Statistical Computing, Vienna, Austria) with the TWANG and SURVEY packages was employed for the analysis.

**References**

1. Harder VS, Stuart EA, Anthony JC. Propensity score techniques and the assessment of measured covariate balance to test causal associations in psychological research. Psychol Methods 2010;15:234-49.

2. Pirracchio R, Petersen ML, van der Laan M. Improving propensity score estimators' robustness to model misspecification using super learner. American journal of epidemiology 2015;181:108-19.

3. McCaffrey DF, Ridgeway G, Morral AR. Propensity score estimation with boosted regression for evaluating causal effects in observational studies. Psychol Methods 2004;9:403-25.

4. McCaffrey DF, Griffin BA, Almirall D, Slaughter ME, Ramchand R, Burgette LF. A tutorial on propensity score estimation for multiple treatments using generalized boosted models. Stat Med 2013;32:3388-414.

5. Robins JM, Hernan MA, Brumback B. Marginal structural models and causal inference in epidemiology. Epidemiology 2000;11:550-60.

6. Breiman L, Friedman JH, Olshen RA, Stone CJ. Classification and Regression Tree. 1983.

7. Wooldridge JM. Econometric analysis of cross section and panel data MIT Press. Cambridge, MA 2002;108.

8. Holland PW. Statistics and Causal Inference. Journal of the American Statistical Association 1986;81:945-60.

9. Rosenbaum PR, Rubin DB. Reducing Bias in Observational Studies Using Subclassification on the Propensity Score. Journal of the American Statistical Association 1984;79:516-24.

10. Ho DE, Imai K, King G, Stuart EA. MTAChing as Nonparametric Preprocessing for Reducing Model Dependence in Parametric Causal Inference. Political Analysis 2007;15:199-236.

11. Stuart EA. MTAChing methods for causal inference: A review and a look forward. Stat Sci 2010;25:1-21.

12. Rubin DB. Using Propensity Scores to Help Design Observational Studies: Application to the Tobacco Litigation. Health Services and Outcomes Research Methodology 2001;2:169-88.
